# Manipulation of Optical Transmittance by Ordered-Oxygen-Vacancy in Epitaxial LaBaCo_2_O_5.5+δ_ Thin Films

**DOI:** 10.1038/srep37496

**Published:** 2016-11-23

**Authors:** Sheng Cheng, Jiangbo Lu, Dong Han, Ming Liu, Xiaoli Lu, Chunrui Ma, Shengbai Zhang, Chonglin Chen

**Affiliations:** 1 School of Electronic and Information Engineering, Xi’an Jiaotong University, Xi’an, 710049, China; 2 State Key Laboratory of Luminescence and Applications, Changchun Institute of Optics, Fine Mechanics and Physics, Chinese Academy of Sciences, Changchun, 130033, P.R. China; 3 State Key Discipline Laboratory of Wide Band Gap Semiconductor Technology, School of Microelectronics, Xidian University, Xi’an, 710071, P.R. China; 4 State Key Laboratory for Mechanical Behavior of Materials, Xi’an Jiaotong University, Xi’an, 710049, P.R. China; 5 Department of Physics, Applied Physics, & Astronomy, Rensselaer Polytechnic Institute, Troy, NY 12180, USA; 6 Department of Physics and Astronomy, University of Texas at San Antonio, TX 78249, USA; 7 The Texas Center for Superconductivity, University of Houston, Houston, Texas 77204, USA

## Abstract

Giant optical transmittance changes of over 300% in wide wavelength range from 500 nm to 2500 nm were observed in LaBaCo_2_O_5.5+δ_ thin films annealed in air and ethanol ambient, respectively. The reduction process induces high density of ordered oxygen vacancies and the formation of LaBaCo_2_O_5.5_ (δ = 0) structure evidenced by aberration-corrected transmission electron microscopy. Moreover, the first-principles calculations reveal the origin and mechanism of optical transmittance enhancement in LaBaCo_2_O_5.5_ (δ = 0), which exhibits quite different energy band structure compared to that of LaBaCo_2_O_6_ (δ = 0.5). The discrepancy of energy band structure was thought to be the direct reason for the enhancement of optical transmission in reducing ambient. Hence, LaBaCo_2_O_5.5+δ_ thin films show great prospect for applications on optical gas sensors in reducing/oxidizing atmosphere.

Perovskite cobaltites have been paid considerable attention of research in the past two decades due to their unique physical properties, such as magnetic, electronic and optical properties, which resulting from the strong correlation and interactions of charge, orbital, spin and photon^1^,^2^,^3^,^4^,^5^,^6^,^7^. Especially, the remarkable mixed conductivity and catalytic properties at high temperature and exciting magnetic and electrical transport properties at low temperature have been intensively studied by scientists and technicians^8^,^9^. All these excellent physical properties have encouraged people to design lots of kinds of functional devices, including solid oxide fuel cells, energy harvest devices, electrical and chemical sensors, etc^10^,^11^,^12^,^13^. Especially, LaBaCo_2_O_5.5+δ_ (LBCO) cobaltite system with three various phase structures of A-site order, nano-area order and disorder, has excited various unique physical properties and phenomena, such as various oxidation states (Co^2+^/Co^3+^/Co^4+^), complex spin state configurations and giant MR effect, etc^14^,^15^,^16^. In order to further investigate the physical properties in such complex cobaltite system, we have fabricated the high quality epitaxial thin films on various substrates and studied its microstructure and physical properties. The epitaxial LBCO thin films exhibit several impressing improvement of physical properties, for example, a much larger magnetoresistance value was observed on epitaxial thin films than those from its bulk material at low temperatures; besides, LBCO thin films possess extraordinary sensitivity to reducing/oxidizing environments, especially an exceedingly fast redox reaction at high temperature^17^,^18^,^19^,^20^,^21^,^22^,^23^,^24^,^25^,^26^,^27^,^28^. Therefore, LBCO thin films are promising candidates for the development of the gas sensors in reducing/oxidizing environments. Until now, the research on the gas sensing properties of the mixed-conductive LBCO system is still at the initial stage. The further study on the gas sensing properties of the LBCO thin films is necessary. In our recent study, the LBCO films exhibited good gas sensing properties at a fairly low temperature of 375 °C, which have the potential for the development of the electronic gas sensor devices by using the resistance switching^13^. However, it is well known that the conventional electronic gas sensors have several defects, such as easily affected by electromagnetic noise and potential safety hazard in flammable gases. The optical gas sensors detect the gases only by identifying the changes of its optical properties, such as transmittance or absorbance^29^,^30^. Therefore, the research on optical gas sensors is becoming more and more important considering the public safety and energy security.

In this paper, the LBCO thin films show the potential application as the optical gas sensor. We find out that the LBCO optical gas sensors can be used to detect the ethanol gas by the transmittance changes of the LBCO films between Air and ethanol vapor gas with obviously high optical sensitivity. Moreover, the ordered oxygen vacancies in LBCO films have been directly measured and the mechanism of optical transmittance enhancement induced by ordered oxygen vacancies has been explored.

## Results

### Gas Sensing and Optical Transmittance Properties

High crystalline quality of epitaxial LBCO thin films have been fabricated on double-polished (La,Sr)(Al,Ta)O_3_ (LSAT) substrates, as shown in the Supplementary Figure S1. The FWHM (full width of half maximum) value for rocking curve measurement from (002) reflection of the LBCO film is about 0.06° with the interface relationship of cube on cube. In order to investigate its optical gas sensing properties of the LBCO thin films, the transmittance changes between air and ethanol vapor gas have been measured. Since there are still no real-time monitoring analysis systems in the commercial market for the changes of optical transmittances between air and ethanol gas, we can’t obtain the response and recovery time as well as *in-situ* optical transmittance change of LBCO thin films. However, the response and recovery times of LBCO resistance gas sensor can indirectly show its response and recovery times of the LBCO optical gas sensor. As shown in Supplementary Figure S2 and Supplementary Figure S3, all these results revealed that the epitaxial LBCO thin films present very reliable, reproducible and fast response(recovery) behavior when ethanol vapor gas is in (out) in the concentration of 10 ppm to 2000 ppm at the optimum working temperature of 375 °C. That is to say the optical gas sensitivity of LBCO thin films can be equivalently obtained through measuring the transmittance spectrum of two LBCO/LSAT samples which were annealed in air and ethanol vapor respectively before optical measurement.

**Figure 1:**
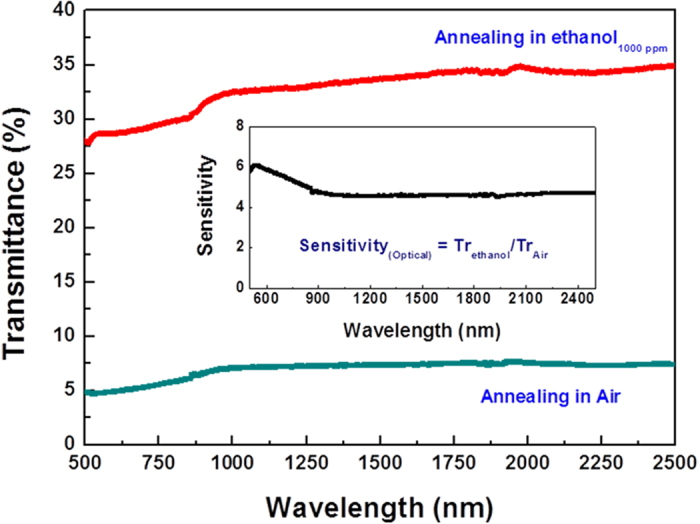
Transmittance spectrum of the LBCO films annealed at air and 1000 ppm ethanol vapor exposure at 375 °C, respectively.

Figure 1 is the transmittance spectrum of the LBCO thin films on (001) LSAT substrates annealed in air and ethanol vapor gas with the concentration of 1000 ppm (Ethanol_1000ppm_) at 375 °C, respectively. It can be clearly seen that the transmittance is obviously enhanced after the LBCO films annealed in Ethanol_1000ppm_ in a wide wavelength range of 500 nm–2500 nm. The optical gas sensitivity has been calculated using the formula as follows:


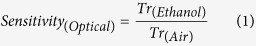


where *Tr*_*(Ethanol)*_ and *Tr*_*(Air)*_ is transmittance of the LBCO films annealed in Air and Ethanol, respectively. As shown in the inset of the Fig. 1, the optical gas sensitivity is 4.6–6.08 in the spectrum range of 500 nm–1000 nm and ~4.6 in the spectrum range of 1000 nm–2500 nm, which indicate that the LBCO films have the great potential of the application as optical gas sensors in wide spectrum ranged from 500 nm to 2500 nm.

### Ordered Oxygen Vacancies Structure

**Figure 2:**
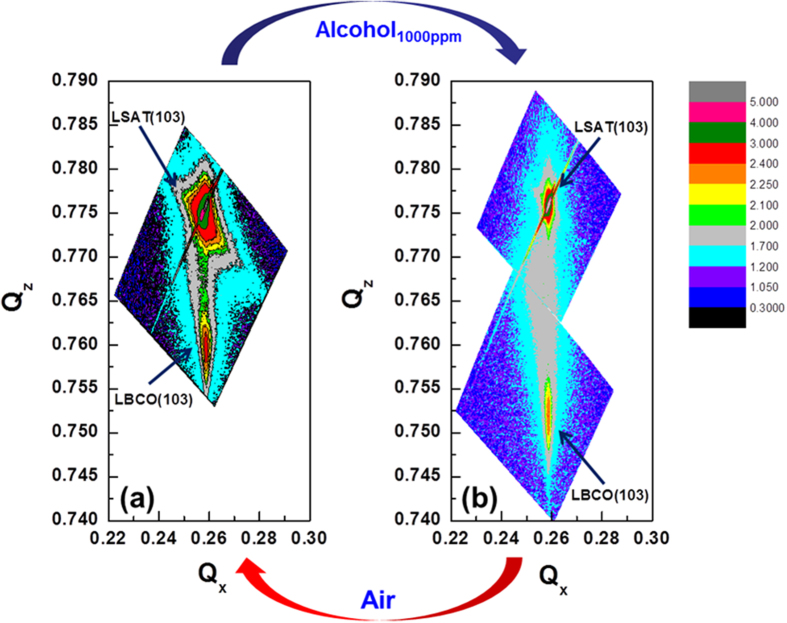
Reciprocal space mapping taken around (103) reflections of the LBCO films annealed at (**a**) air, and (**b**) 1000 ppm ethanol vapor exposure at 375 °C, respectively.

In order to systematically study the possible mechanism of the transmittance enhancement of the LBCO films in reducing environment, the high-resolution theta-2theta and reciprocal space mapping (RSMs) technique are performed to determine the phase structures and lattice parameters for both the LBCO films annealed in Air (Air sample) and Ethanol_1000ppm_ (Ethanol_1000ppm_ sample) at 375 °C. Both the films are *c*-axis orientation without any other second phase, revealed that the oxidation-reduction reaction didn’t change its phase structure. Figure 2(a,b) is the asymmetric (013) reflections from both the Air sample and the Ethanol_1000ppm_ sample, respectively. The in-plane and out-of-plane lattice parameters of the air and Ethanol_1000ppm_ samples can be calculated from the RSMs and listed in Table 1. It is shown that the in-plane lattice parameters of the Air sample and the Ethanol_1000ppm_ sample are equal to that of the LSAT substrate, which revealing that both of them are fully strained films. However, the out-of-plane lattice parameters of the two films have a significantly change, *c*_Air_ = 3.946 Å and *c*_Ethanol1000ppm_ = 4.000 Å. The tetragonality (*c/a*) is 1.020 and 1.034 for the Air and Ethanol_1000ppm_ samples, respectively. Tetragonality increases after the LBCO films annealed in Ethanol_1000ppm_ environments. It is well known that the high density of oxygen vacancies will be induced in LBCO thin films when they areexposed to reducing gas, such as ethanol vapor. The chemical processing can be expressed as follows^13^:


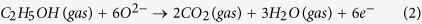


Moreover, a larger number of oxygen vacancies formatted in the LBCO films will induce the increase of *c*-axis lattice parameter, which is in agreement with our RSM measurements^31^,^32^,^33^,^34^.

**Figure 3:**
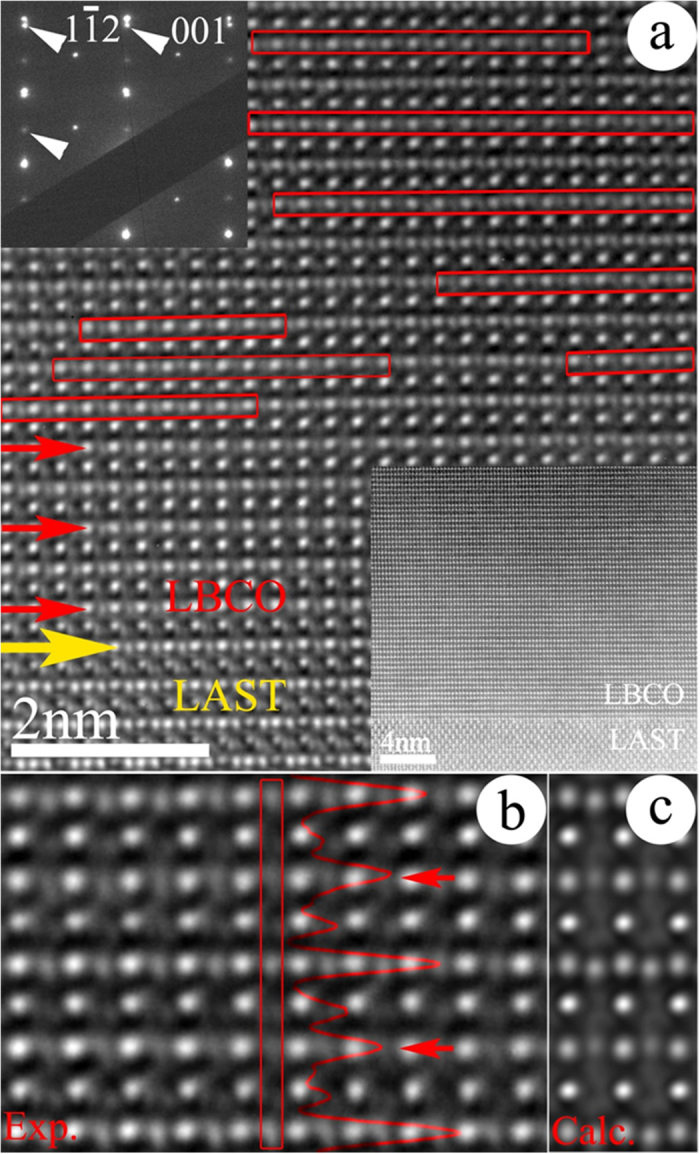
(**a**) High resolution transmission electron microscopy image using the NCSI technique of the LBCO thin film and LSAT substrate. Atom sites appear bright on a dark background. The yellow arrow denotes the interface. The red rectangles and arrows denote the oxygen deficient layers. The spherical aberration coefficient was adjusted for C_S_ = −15 μm, and an over focus of ΔZ = +5 nm was used. Inset in the upper left corner shows the selected area diffraction patterns from the interface area of film and substrate. Inset in the lower right corner shows a lower magnification HAADF-STEM image of the film and substrate. (**b**) Magnified typical image using NCSI technique of LBCO film from thin area indicating the ordered oxygen vacancy structure. (**c**) Calculated image of the LaBaCo_2_O_5.5_ (spherical aberration coefficient C_S_ = −15 μm; specimen thickness: 3 nm; defocus ΔZ = +3 nm; convergence angle: 0.2 mrad; defocus spread: 2.8 nm). The red rectangle shows the region where the intensity line profile analysis was carried out, the red curve shows the corresponding intensity trace. The red arrows indicate the oxygen vacancy layers.

In order to observe the oxygen vacancies’ structure in LBCO films, high resolution transmission electron microscopy image using the negative spherical aberration Cs imaging (NCSI) technique^35^,^36^,^37^ was employed. Previous research reported that LaBaCo_2_O_5.5_ and LaBaCo_2_O_5_ with oxygen vacancies could be induced one after another when LBCO epitaxial thin films were annealed at H_2_. In our case, however, LaBaCo_2_O_5_ was ruled out because LaBaCo_2_O_5.5_ to LaBaCo_2_O_5_ conversion is accompanied with giant decrease of thin films’ resistance^24^,^25^ which was not observed in our experiments, as shown in Supplementary Figure S2 and Figure S3. Thus it is most likely that the only LaBaCo_2_O_5.5_ was induced in ethanol vapor. NCSI images have been used to prove the existence of ordered oxygen vacancy structure in the Ethanol_1000ppm_ LBCO sample, as shown in Fig. 3(a) taken along [110] zone axis. The yellow arrow indicates the interface between the LBCO thin film and LSAT substrate. The alternating weak contrast of oxygen columns in Co-O layers indicated by red arrows and rectangles in the LBCO film results from the ordering of oxygen vacancies in every other Co-O planes. This observation suggests that a large number of ordered oxygen vacancies can be generated in the LBCO thin film by reduction treatment. It is noted that the ordered oxygen vacancy structure may not be obviously observed in some small region in Fig. 3(a), the reason could be the nonhomogeneous reduction and the overlaps of oxygen deficient and stoichiometric columns. The inset in the upper left corner shows the selected area diffraction patterns taken from the interface area of film and substrate along the [110] zone axis of film. The diffraction spots from the film are labeled by white arrows. Weak diffraction spots at the midpoints between two strong spots can be seen, demonstrating the doubling of the unit cells along [001] orientation. The inset in the lower right corner shows a lower magnification aberration-corrected high angle annular dark field (HAADF) scanning transmission electron microscopy (STEM) image of the LBCO thin film and LSAT substrate viewed along [110] zone axis. Figure 3(b) shows the magnified typical experimental NCSI image of LBCO film from thin area indicating the ordered oxygen vacancy structure. An intensity trace indicated by red curve is displayed in Fig. 3(b), taken from the region indicated by red rectangle. The red arrows indicate the oxygen deficient layers. Figure 3(c) shows the calculated image with the formula LaBaCo_2_O_5.5_. It can be seen that the calculated image matches with the experimental image indicating the oxygen concentration of LBCO thin film after reduction treatment is close to LaBaCo_2_O_5.5._

### Theoretical Modeling and Calculations

The oxygen vacancies in the LBCO films can significantly affect these properties, including the conductivity and optical property. As we know, the band structure of LBCO around the Fermi level is determined by the hybridization of Co ions and O ions. The band structure would be remarkably changed by massively removing the O ions since the oxygen vacancies not only change the charge state of Co ion locally, but also alter the local spin state and overall spin configuration of Co ions. For example, under Air ambient, the concentration of oxygen vacancies decreases and parts of Co^3+^ have been changed into Co^4+^, which induce the resistance decreases. Once the atmosphere has been changed from Air into ethanol vapor gas, the concentration of oxygen vacancies increases and parts of Co^4+^ have been changed into Co^3+^, which induce the resistance increases^32^,^33^. To clearly understand the mechanism of optical transmission enhancement induced by large numbers of ordered oxygen vacancies, the first-principle calculations based on the Density functional theory (DFT) have been performed to obtain the optical properties for the LBCO films without and with the ordered oxygen vacancies (LaBaCo_2_O_6_ and LaBaCo_2_O_5.5+δ_). The value of δ, which reflects the oxygen vacancy concentration in the LBCO film, is varied between 0 and 0.5 based on whether the film is annealed in ethanol ambient. For simplicity, we assumed that δ = 0 if the film was annealed in ethanol gas, namely LaBaCo_2_O_5.5_. Note that, the optimized geometry of LaBaCo_2_O_5.5_ is in good agreement with the NCSI images, as shown in Fig. 3. In LaBaCo_2_O_6_, the calculated total energy with antiferromagnetic (AF) phase is slightly smaller (87 meV/molecular) than that with ferromagnetic (FM) phase. However, the LaBaCo_2_O_5.5_ with AF phase is much stable with lowering the energy of 0.45 eV/molecular, compared to the LaBaCo_2_O_5.5_ with FM phase. This phenomenon can be explained by the Double-exchange/Superexchange mechanism. The calculation results show the magnetic moment of Co^3+^ ion in LaBaCo_2_O_5.5_ is about 3.0 *μ*_*B*_ and indicate the *e*_*g*_ and *t*_*2g*_ levels of Co^3+^ ion are all occupied by the spin electrons. No spin electron can transfer by hopping through intermediary O ion between Co^3+^ ions, as explained in the Superexchange mechanism. It would lower the energy of LaBaCo_2_O_5.5_ with AF phase. In LaBaCo_2_O_6_, one more electron in one of two Co^3+^ ions is oxidized. As a result, one empty *e*_*g*_ level of Co^4+^ ion is allowed to transfer the spin electron through intermediary O ion between Co^4+^ ion and Co^3+^ ion, which would lower the energy of LaBaCo_2_O_6_ with FM phase as explained in the Double-exchange mechanism. Therefore, in the LBCO film annealed in the air, it indicates the coexistence of AF and FM phases at room temperature, while the LBCO film with oxygen vacancies annealed in the ethanol vapor gas is AF-phase dominant.

**Figure 4:**
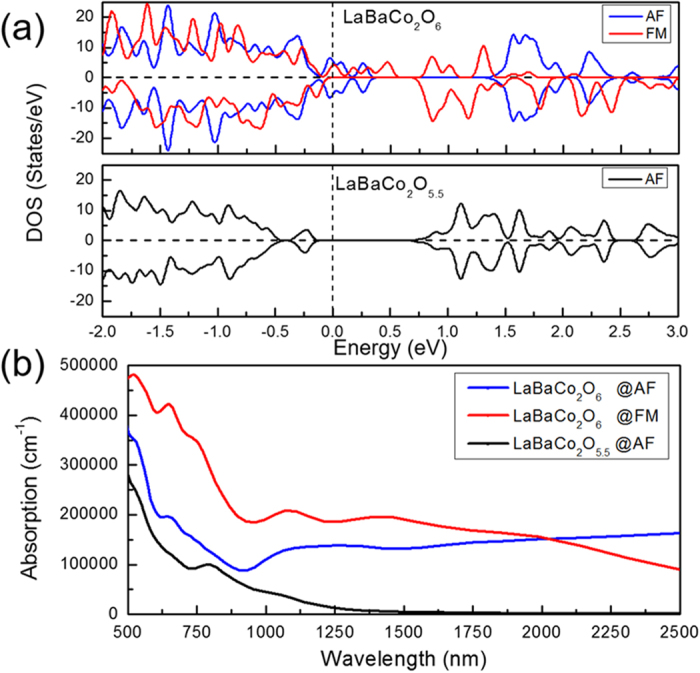
(**a**) The Density of states (DOS) of LaBaCo_2_O_6_ with both antiferromagnetic and ferromagnetic phases and LaBaCo_2_O_5.5_ with antiferromagnetic phase. The positive value of DOS stands for spin up and the negative value of DOS stands for spin down. The dash line at zero stands for Fermi level. (**b**) The calculated optical absorption of LaBaCo_2_O_6_ with both antiferromagnetic and ferromagnetic phases and LaBaCo_2_O_5.5_ with antiferromagnetic phase.

The optical transmittance enhancement of the LBCO films grown in the ethanol vapor gas can be considered to the response of reflection and absorption. With no obvious reflection difference is found, the transmittance enhancement of LBCO films is mainly attributed to the optical absorption. Figure 4(a) shows the density of states (DOS) of the two-type LBCO films. It can be found that the LaBaCo_2_O_6_ with both AF and FM phases has no band gap, which signifies the strong optical absorption. But the LaBaCo_2_O_5.5_ shows a clear gap with the energy gap of about 1 eV, which indicates no photon with the wavelength shorter than nearly 1250 nm can be absorbed. The calculated optical absorptions are shown in Fig. 4(b). It clearly shows that the optical absorption of LaBaCo_2_O_6_ is significantly stronger than that of LaBaCo_2_O_5.5_ during the entire wavelength range from 500 nm to 2500 nm. Correspondingly, the LaBaCo_2_O_5.5_ starts to absorb the light when the wavelength of light is shorter than 1250 nm. The contribution of optical absorption in LaBaCo_2_O_6_ can be separated into two parts. At the long wavelength range, as shown around from 2000 nm to 2500 nm, the optical absorption of AF-phase LaBaCo_2_O_6_ is dominant because of the large DOS of AF phase near the Fermi level. The main optical absorption comes from the FM-phase LaBaCo_2_O_6_ at the short wavelength range from 500 nm to 1500 nm since no band gap exist in FM-phase LaBaCo_2_O_6_. Compared to FM-phase LaBaCo_2_O_6_, The weak absorption of AF-phase LaBaCo_2_O_6_ is because electron with the energy of from 500 nm to 1500 nm is insufficient in energy to be excited from the half occupied valence band to its upper conduction band. Overall, the optical absorption of LaBaCo_2_O_5.5_ is much less than that of LaBaCo_2_O_6_. Note that, by considering the band-gap underestimation in DFT calculation, the spectrum of optical absorption should blue shift, which matches the transmittance spectrum of LBCO films even better. Therefore, it should be a direct cause resulted in the transmittance enhancement of the LBCO films in reducing environment without the deterioration of the crystal structure.

In summary, giant optical transmittance enhancement of over 300% in the wavelength range from 500 nm to 2500 nm has been observed in LaBaCo_2_O_5.5_ films. The reduction process induces ordered oxygen vacancies in LaBaCo_2_O_5.5_ structure evidenced by aberration-corrected transmission electron microscopy. Moreover, the mechanism of the transmission enhancement has been explored by using the first-principles calculations, revealing LaBaCo_2_O_5.5_ exhibits quite different energy band structure compared to that of LaBaCo_2_O_6_ and the discrepancy of energy band structure was thought to be the direct reason. It is suggested that the LaBaCo_2_O_5.5+δ_ thin films have great potential to develop the wide spectrum optical gas sensors in reducing/oxidizing environments.

## Methods

### Film preparation

A KrF excimer pulsed laser deposition system with a wavelength 248 nm has been performed to fabricate the LBCO thin films on (001) (La,Sr) (Al,Ta)O_3_ (LSAT) single-crystalline substrates. The growth condition was selected under an oxygen pressure of 250 mTorr at 700 °C. The laser energy density is about 2.0 J/cm^2^ at 5 Hz. After finishing the growth of the LBCO thin film, it was annealed at 700 °C for 15 min in pure oxygen (200 Torr), then cooled down to room temperature at the rate of 30 °C/min.

### Structural, electrical and optical characterization

The crystalline quality and epitaxial behavior of the LBCO thin films were characterized by high-resolution x-ray diffraction (HRXRD) using PANalytical X’Pert MRD. The [110] cross-sectional specimens for high-resolution transmission electron microscopy investigation were prepared with the “lift-out” technique using an FEI Helios 600i FIB/SEM dual-beam system. The final thinning of the FIB specimens was carried out with Gatan precision ion polishing system (PIPS). High-resolution transmission electron microscopy investigations using negative Cs imaging technique (NCSI) were carried out on an FEI TITAN G2 60–300 microscope with a CS corrector for the objective lens, operated at 300 kV. The atomic resolution high angle annular dark field scanning transmission electron microscopy (HAADF-STEM) investigations were carried out on a JEOL ARM 200 F with a probe aberration corrector, operated at 300 kV. The atomic resolution high angle annular dark field scanning transmission electron microscopy (HAADF-STEM) investigations and selected area electron diffraction (SAED) were carried out on a JEOL ARM 200 F with a probe aberration corrector, operated at 200 kV. All images were calculated with the electron beam parallel to the [110] zone axis using the MacTempas software package^38^. The ethanol sensing properties of the LBCO thin films were systematically studied using the GS-1TP intelligent gas sensing analysis system under the concentration between 10 ppm to 2000 ppm in the temperature ranged from 250 °C to 450 °C^39^. And transmission measurement was taken by using a Perkin-Elmer Lambda 950 spectrophotometer. The optical transmittance from 500 nm to 2500 nm of a 10 mm × 10 mm air annealed LBCO/LSAT(001) sample, a 10 mm × 10 mm Ethanol annealed LBCO/LSAT(001) sample and a 10 mm × 10 mm bare LSAT(001) substrate was measured respectively, and the transmittance of bare substrate was minus from that of thin films to obtain the net transmittance of thin films.

### Theory section

First-principles calculations based on the density-functional theory (DFT)^40^,^41^ were carried out using the VASP codes^42^. The generalized gradient approximation (GGA)^43^ with the Perdew-Burke-Ernzerhof^44^ functional and projector augmented wave basis^45^ are employed. The perovskite cell of LaBaCo2O6 includes 10 atoms and the lattice parameters is 3.868 Å for a, 3.868 Å for b and 7.892 Å for c, respectively, which agrees with the experimental value. The atomic cell of LaBaCo_2_O_5.5_ is the 2 × 1 × 1 supercell of LaBaCo_2_O_6_ with removing one oxygen in Co-O plane. The cut off energy and Monkhorst-Pack k-point mesh grid are 600 eV and 3 × 3 × 3, respectively. The atomic structures were optimized until the Hellman-Feynman forces on all atoms were smaller than 0.015 eV/Å.

## Additional Information

**How to cite this article**: Cheng, S. *et al*. Manipulation of Optical Transmittance by Ordered-Oxygen-Vacancy in Epitaxial LaBaCo_2_O_5.5+δ_ Thin Films. *Sci. Rep.* **6**, 37496; doi: 10.1038/srep37496 (2016).

**Publisher's note:** Springer Nature remains neutral with regard to jurisdictional claims in published maps and institutional affiliations.

## Supplementary Material

Supplementary Information

## Figures and Tables

**Table 1:** In-plane and out-of-plane lattice parameters and tetragonality of the LBCO films annealed in air and Ethanol_1000 ppm_ environments.

Different ambient gases	Lattice Parameters (in-plane)	Lattice Parameter (out-of-plane)	Tetragonality (c/a)
Air	3.868 Å	3.946 Å	1.020
Ethanol_1000ppm_	3.868 Å	4.000 Å	1.034

## References

[b1] Taskin A. A., Lavrov A. N. & Ando Y. Achieving fast oxygen diffusion in perovskites by cation ordering. Appl Phys Lett 86 (2005).

[b2] Kim G. . Rapid oxygen ion diffusion and surface exchange kinetics in PrBaCo_2_O_5+x_ with a perovskite related structure and ordered A cations. J Mater Chem 17, 2500–2505 (2007).

[b3] Fauth F. . Interplay of structural, magnetic and transport properties in the layered Co-based perovskite LnBaCo_2_O_5_ (Ln = Tb, Dy, Ho). Eur Phys J B 21, 163–174 (2001).

[b4] De Souza R. A. & Kilner J. A. Oxygen transport in La_1–x_Sr_x_Mn_1–y_Co_y_O_3±δ_ perovskites - Part I. Oxygen tracer diffusion. Solid State Ionics 106, 175–187 (1998).

[b5] Luo G. P. . Electrical and magnetic properties of La_0.5_Sr_0.5_CoO_3_ thin films. Appl Phys Lett 76, 1908–1910 (2000).

[b6] Yuan Z. . Epitaxial behavior and transport properties of PrBaCo_2_O_5_ thin films on (001) SrTiO_3_. Appl Phys Lett 90 (2007).

[b7] Fauth F., Suard E. & Caignaert V. Intermediate spin state of Co^3+^ and Co^4+^ ions in La_0.5_Ba_0.5_CoO_3_ evidenced by Jahn-Teller distortions. Phys Rev B 65 (2002).

[b8] Post M. L., Tunney J. J., Yang D., Du X. & Singleton D. L. Material chemistry of perovskite compounds as chemical sensors. Sensor Actuat B-Chem 59, 190–194 (1999).

[b9] Wang D., Tunney J. J., Du X. M., Post M. L. & Gauvin R. Thermal stability of SrFeO_3_/Al_2_O_3_ thin films: Transmission electron microscopy study and conductometric sensing response. J Appl Phys 104 (2008).

[b10] Jacobson A. J. Materials for Solid Oxide Fuel Cells. Chem Mater 22, 660–674 (2010).

[b11] Wang S., Verma A., Yang Y. L., Jacobson A. J. & Abeles B. The effect of the magnitude of the oxygen partial pressure change in electrical conductivity relaxation measurements: oxygen transport kinetics in La_0.5_Sr_0.5_CoO_3−δ_. Solid State Ionics 140, 125–133 (2001).

[b12] Kim G. . Oxygen exchange kinetics of epitaxial PrBaCo_2_O_5+δ_ thin films. Appl Phys Lett 88 (2006).

[b13] Liu M. . Gas Sensing Properties of Epitaxial LaBaCo_2_O_5.5+δ_ Thin Films. Sci Rep 5, 10784 (2015). 26146369 10.1038/srep10784 PMC4491845

[b14] Nakajima T., Ichihara M. & Ueda Y. New A-site ordered perovskite cobaltite LaBaCo_2_O_6_: Synthesis, structure, physical property and cation order-disorder effect. Journal of the Physical Society of Japan 74, 1572–1577 (2005).

[b15] Rautama E. L. . Cationic ordering and microstructural effects in the ferromagnetic perovskite La_0.5_Ba_0.5_CoO_3_: Impact upon magnetotransport properties. Chem Mater 20, 2742–2750 (2008).

[b16] Rautama E. L. . New Member of the “112” Family, LaBaCo_2_O_5.5_: Synthesis, Structure, and Magnetism. Chem Mater 21, 102–109 (2009).

[b17] Liu J. . Epitaxial Nature and Transport Properties in (LaBa)Co_2_O_5+δ_ Thin Films. Chem Mater 22, 799–802 (2010).

[b18] Liu J. . PO_2_ dependant resistance switch effect in highly epitaxial (LaBa)Co_2_O_5+δ_ thin films. Appl Phys Lett 97, 094101 (2010).

[b19] Liu M. . Magnetic and electrical transport properties of LaBaCo_2_O_5.5+δ_ thin films directly integrated on Si (001). Mater Lett 109, 143–145 (2013).

[b20] Liu M. . Magnetic and transport properties of epitaxial (LaBa)Co_2_O_5.5+δ_ thin films on (001) SrTiO_3_. Appl Phys Lett 96, 3 (2010).

[b21] Liu J. . Ultrafast oxygen exchange kinetics on highly epitaxial PrBaCo_2_O_5+δ_ thin films. Appl Phys Lett 100, 193903 (2012).

[b22] Ma C. R. . Magnetic and Electrical Transport Properties of LaBaCo_2_O_5.5+δ_ Thin Films on Vicinal (001) SrTiO_3_ Surfaces. Acs Appl Mater Inter 5, 451–455 (2013). 10.1021/am302553y 23270544

[b23] Liu M. . Strain-induced anisotropic transport properties of LaBaCo_2_O_5.5+δ_ thin films on NdGaO_3_ substrates. ACS Appl Mater Interfaces 6, 8526–8530 (2014). 24824560 10.1021/am502448k

[b24] Bao S. . Ultrafast atomic layer-by-layer oxygen vacancy-exchange diffusion in double-perovskite LnBaCo_2_O_5.5+δ_ thin films. Sci Rep 4, 4726 (2014). 24751601 10.1038/srep04726 PMC3994446

[b25] Wang H. B. . Ultrafast chemical dynamic behavior in highly epitaxial LaBaCo_2_O_5+δ_ thin films. J. Mater. Chem. C 2, 5660–5666 (2014).

[b26] Ma C. R. . Interface effects on the electronic transport properties in highly epitaxial LaBaCo_2_O_5.5+δ_ films. ACS Appl Mater Interfaces 6, 2540–2545 (2014). 24467686 10.1021/am404951v

[b27] Zou Q. . Step Terrace Tuned Anisotropic Transport Properties of Highly Epitaxial LaBaCo_2_O_5.5+δ_ Thin Films on Vicinal SrTiO_3_ Substrates. Acs Appl Mater Inter 6, 6704–6708 (2014). 10.1021/am500422j 24716582

[b28] He J. . Self-patterned Nano Structures in Structurally Gradient Epitaxial La_0.5_Ba_0.5_CoO_3_ Films. Thin Solid Films 519, 4371–4376 (2011).

[b29] Nam H. J., Sasaki T. & Koshizaki N. Optical CO gas sensor using a cobalt oxide thin film prepared by pulsed laser deposition under various argon pressures. J Phys Chem B 110, 23081–23084 (2006). 17107147 10.1021/jp063484f

[b30] Zaharieva J., Milanova M. & Todorovsky D. SiO_2_/polyester hybrid for immobilization of Ru(II) complex as optical gas-phase oxygen sensor. J Mater Chem 21, 4893–4903 (2011).

[b31] Jeen H. . Reversible redox reactions in an epitaxially stabilized SrCoO_x_ oxygen sponge. Nature Materials 12, 1057–1063 (2013). 23975056 10.1038/nmat3736

[b32] Kim Y. M. . Probing oxygen vacancy concentration and homogeneity in solid-oxide fuel-cell cathode materials on the subunit-cell level. Nature Materials 11, 888–894 (2012). 22902896 10.1038/nmat3393

[b33] Donner W. . Epitaxial Strain-Induced Chemical Ordering in La_0.5_Sr_0.5_CoO_3−δ_ Films on SrTiO_3_. Chem Mater 23, 984–988 (2011).

[b34] Petrie J. R. . Strain Control of Oxygen Vacancies in Epitaxial Strontium Cobaltite Films. Adv Funct Mater 26, 1564–1570 (2016).

[b35] Jia C. L., Lentzen M. & Urban K. Atomic-resolution imaging of oxygen in perovskite ceramics. Science 299, 870–873 (2003). 12574624 10.1126/science.1079121

[b36] Jia C. L. & Urban K. Atomic-resolution measurement of oxygen concentration in oxide materials. Science 303, 2001–2004 (2004). 15044799 10.1126/science.1093617

[b37] Jia C. L., Lentzen M. & Urban K. High-resolution transmission electron microscopy using negative spherical aberration. Microsc. microanal. 10, 174–184 (2004). 15306044 10.1017/S1431927604040425

[b38] Okeefe M. A. & Kilaas R. Advances in High-Resolution Image Simulation. Scanning Microscopy, 225–244 (1988).

[b39] Guo Y., Xiao Y. P., Zhang L. M. & Song Y. F. Fabrication of (Calcein-ZnS)_n_ Ordered Ultrathin Films on the Basis of Layered Double Hydroxide and Its Ethanol Sensing Behavior. Ind Eng Chem Res 51, 8966–8973 (2012).

[b40] Hohenberg P. & Kohn W. Inhomogeneous Electron Gas. Phys Rev B 136, B864-+ (1964).

[b41] Kohn W. & Sham L. J. Self-Consistent Equations Including Exchange and Correlation Effects. Physical Review 140, 1133 (1965).

[b42] Kresse G. & Furthmuller J. Efficient iterative schemes for ab initio total-energy calculations using a plane-wave basis set. Phys Rev B 54, 11169–11186 (1996). 10.1103/physrevb.54.11169 9984901

[b43] Perdew J. P. & Yue W. Accurate and Simple Density Functional for the Electronic Exchange Energy - Generalized Gradient Approximation. Phys Rev B 33, 8800–8802 (1986). 10.1103/physrevb.33.8800 9938293

[b44] Perdew J. P., Burke K. & Ernzerhof M. Generalized gradient approximation made simple. Phys Rev Lett 77, 3865–3868 (1996). 10062328 10.1103/PhysRevLett.77.3865

[b45] Blochl P. E. Projector Augmented-Wave Method. Phys Rev B 50, 17953–17979 (1994). 10.1103/physrevb.50.17953 9976227

