# Supplementary material for: Manipulation of Optical Transmittance by Ordered-Oxygen-Vacancy in Epitaxial LaBaCo2O5.5+δ Thin Films
Source: Sci Rep. 2016 Nov 23;6:37496. doi: 10.1038/srep37496 (PMC5120332; doi:10.1038/srep37496)
Supplement: Supplementary Information [file srep37496-s1.doc]

Supplementary material

**Manipulation of Optical Transmittance by Ordered-Oxygen-Vacancy in Epitaxial LaBaCo_2_O_5.5+δ_ Thin Films**

Sheng Cheng^1^,[[1]](#footnote-2)), Jiangbo Lu^1,a)^, Dong Han^2,a)^, Ming Liu^1^,[[2]](#footnote-3)), Xiaoli Lu^3^, Chunrui Ma^4^, Shengbai Zhang^5^, Chonglin Chen^6,7^

^1^ School of Electronic and Information Engineering, Xi’an Jiaotong University, Xi’an 710049, China

^2^ State Key Laboratory of Luminescence and Applications, Changchun Institute of Optics, Fine Mechanics and Physics, Chinese Academy of Sciences, Changchun 130033, P. R. China

^3^ State Key Discipline Laboratory of Wide Band Gap Semiconductor Technology, School of Microelectronics, Xidian University, Xi’an710071, P. R. China

^4^ State Key Laboratory for Mechanical Behavior of Materials, Xi’an Jiaotong University, Xi’an 710049, P. R. China

^5^Department of Physics, Applied Physics, & Astronomy, Rensselaer Polytechnic Institute, Troy, NY 12180, USA

^6^Department of Physics and Astronomy, University of Texas at San Antonio, TX 78249, United States

^7^The Texas Center for Superconductivity, University of Houston, Houston, Texas 77204, United States

The phase structure and crystalline quality of the LBCO films have been characterized by the XRD *θ−2θ* scan and rocking curving, *φ* scan. As shown in the Figure S1, it is the XRD *θ−2θ* scanning pattern of the as-grown LBCO films on LSAT substrates. Only the (00*l*) peaks appear in the *θ-2θ* scans for the LBCO films, revealing that the films are *c*-axis oriented growth. The rocking curve measurements from the (002) reflections for the films show that the full width of half maximum (FWHM) is about 0.06°, as shown in the inset (a) of Figure S1, suggesting that the as-grown LBCO films have excellent single-crystalline quality. The *φ* scans measurements have been used to study the in-plane crystallographic relationship between the films and the substrates. The inset (b) of Figure S1 shows the *φ* scans taken around the {101} reflections of the LBCO films and the LSAT substrates. The four-fold symmetry and sharp peaks in the *φ* scan pattern of the LBCO films suggest that the films have excellent epitaxial crystalline quality. Based on the data of the *φ* scans and *θ−2θ* scan, the orientation relationship between the films and the substrates is determined as [100]_LBCO_//[100]_LSAT_ and [001]_LBCO_//[001]_LSAT_.

To investigate the resistance switching gas sensing properties of LBCO thin films, the optimum working temperature, transient response during the change between air and various ethanol vapor concentration have been investigated. Figure S2 is the gas sensing transient response of the LBCO films to 1000 ppm ethanol vapor exposure as a function of working temperature from 250°C to 450°C. It can be clearly seen that the resistance of LBCO thin films increase when exposed to the ethanol vapor gas and decrease when exposed to air. The LBCO thin films can detect ethanol gas very fast at the temperature of 250°C and 300°C. However, their resistance can’t be recovered completely in short time when it exposed to air. Moreover, the LBCO films with the working temperature of 450°C cannot obtain a larger sensitivity (*S*) than that of the LBCO films with the working temperature of 375°C. The *S* can be determined by using the formula *S* = R_g_/R_a_, where R_a_ and R_g_ is resistance of the LBCO film in air and in testing gas atmosphere, respectively. Therefore, the temperature at 375°C is the best operation condition from the four experimental data for the good performance as potential ethanol/reducing gas sensor.

Figure S3(a) is the gas sensing transient response of the LBCO films to various ethanol vapor exposure from 10 ppm to 2000 ppm at 375°C.Most importantly, even at a very low ethanol vapor concentration of 10 ppm, the LBCO films still can detect transient response. It is well known that the response and recovery times for the LBCO thin film as ethanol gas sensors are the two most important quantity factors for gas sensors. The response and recovery times are defined as the time required reaching 90% response (recovery) when the gas is in (out). As shown in Figure S3(b), the response time increases from ~22s (10 ppm) to ~50s (2000 ppm) and the recovery time decreases from ~32s (10 ppm) to ~6s (2000 ppm) with the increase of ethanol vapor concentrations. All these data reveal that the LBCO epitaxial films present a reliable and reproducible response during the change between air and various ethanol vapor concentrations.

**Figure Legends:**

Figure S1. Typical XRD pattern of the as-grown LBCO films grown on (001) LSAT substrates. The inset (a) and the inset (b) is the rocking curve from the (002) diffraction and the *φ* scans taken around the (101) diffraction of the LBCO film, respectively.

Figure S2. Transient resistance responses of the LBCO films on LSAT substrates during the change of air to 1000 ppm ethanol vapor exposure at different temperatures from 250°C to 450°C.

Figure S3 (a) Transient responses, (b) response time and recovery time of the LBCO films during the change of air to various ethanol vapor concentrations at 375°C.

**Figure S1**

**Figure S2**

**Figure S3**

1. These authors contribute equally to this work. [↑](#footnote-ref-2)
2. Electronic mail: [m.liu@mail.xjtu.edu.cn](mailto:m.liu@mail.xjtu.edu.cn) [↑](#footnote-ref-3)
